# Critical Success Factors for Avoiding the Disruption of Assistive Technology Services in the Post-Pandemic Era

**DOI:** 10.3390/healthcare14101277

**Published:** 2026-05-08

**Authors:** Wei Hsu, Shu-Mei Tseng, Ling-Na Shih

**Affiliations:** 1 Department of Health Care Management, National Taipei University of Nursing & Health Sciences, Taipei City 112303, Taiwan; 2 Superintendent Office, Lo-Sheng Hospital, Ministry of Health and Welfare, New Taipei City 242030, Taiwan

**Keywords:** assistive technology (AT), critical success factors (CSFs), COVID-19, disruption of services, multicriteria decision-making (MCDM)

## Abstract

**Background/Objectives:** Individuals with limitations in their daily activities use assistive technology (AT), which helps them restore body structures and functions. During the pandemic, to prevent the spread of infection, health policies have disrupted the traditional delivery mode of AT service, and the lack of preparedness for contingency measures has further caused AT service disruptions, making the continuity of AT services a major challenge. This study explores the critical success factors (CSFs) for preventing AT service interruptions in the post-pandemic era and supporting decision-makers in responding rapidly to similar infectious disease pandemics in the future, while ensuring delivery of high-quality AT services. **Methods:** A systematic literature review was conducted, and then, the multicriteria decision-making (MCDM) model, combined with a decision-making trial and evaluation laboratory (DEMATEL) and an analytic network process (ANP), was applied to stratify complex problems in a structured manner, thereby constructing a multicriteria decision analysis structure for identifying the CSFs for avoiding AT service interruptions in the post-pandemic era. **Results:** The study results revealed that the three most influential direct factors are improving the providers’ telemedicine capabilities, enhancing access to digital AT service support, and establishing a digital AT ecosystem. Indirect factors include addressing resource shortages. **Conclusions:** To avoid repeating past mistakes during future pandemics involving similar infectious diseases, strengthening the telemedicine capabilities of medical staff and ensuring a complete AT service delivery system are the most essential priorities.

## 1. Introduction

Assistive technology (AT) supports individuals with temporary or permanent disabilities, chronic illness, or declining physical and psychosocial functioning. The World Health Organization reported that approximately 1 billion people currently need AT, and this number is expected to exceed 2 billion by 2030 [1]. The Global Alliance of AT Organizations promotes global networks of AT services and information centers and works to expand access and awareness of AT, ensuring that users maintain essential skills and remain active in society [2]. Since the COVID-19 pandemic led to lockdowns, quarantines, and social distancing, infection control policies reduced the number of opportunities for one-on-one AT support, disrupting the continuity of care [3]. In 60–70% of countries, rehabilitation services were interrupted, placing individuals and their families at a greater risk of disability-related challenges [4]. Limited access to AT during the pandemic severely affected users’ daily lives, education, and employment, highlighting the urgent need for resilient AT service delivery systems.

With the advent of infectious disease pandemics, such as COVID-19, enhanced communication has become a key component of intervention strategies. Delays in the delivery of important messages by policymakers can be detrimental to individuals who need AT, especially those with hearing loss [5]. For example, during the coronavirus disease 2019 (COVID-19) pandemic, the use of masks and other personal protective equipment created communication barriers for people with hearing impairment, and these restrictions were even more pronounced in healthcare settings. As a result, the current state of public health response may even exacerbate disparities in access to care [6]. The lack of an effective AT supply management system in Ireland has led to suggestions to provide temporary funding to support the country during the crisis, and experts are optimistic that AT will play a central role in establishing a sustainable supply management system across the country [7]. To prepare for pandemics in the future, we should review AT service delivery and acquisition processes, ensuring high-quality services while providing decision-makers with a reference for future strategy formulation through the results of research and analysis.

Some studies have examined AT services using qualitative methods [3], whereas some have used quantitative methods such as Likert-scale surveys. Most findings focus on disabled people, families, schools and governments. However, fewer studies have combined qualitative and quantitative research and made specific recommendations for hospitals and policy makers while simultaneously considering multiple criteria of AT services. The health policies of various countries have become committed to pandemic prevention; thus, this study combines a qualitative systematic literature review and quantitative multicriteria decision-making (MCDM) methods to fully understand the impact of a pandemic on AT service provision. The decision-making trial and evaluation laboratory (DEMATEL) and analytic network process (ANP) of the MCDM methods are applied to identify CSFs. These methods allow hospital decision-makers and policy makers to rapidly and more efficiently identify these CSFs to avoid AT service interruptions during similar infectious disease outbreaks in the future. The purpose of this study is to explore the key CFSs for preventing the disruption of AT services in the post-pandemic era through a systematic literature search and MCDM, thereby providing decision-makers with rapid solutions to improve the delivery of AT services when encountering similar infectious disease pandemics in the future.

## 2. Literature Review

**Figure 1:**
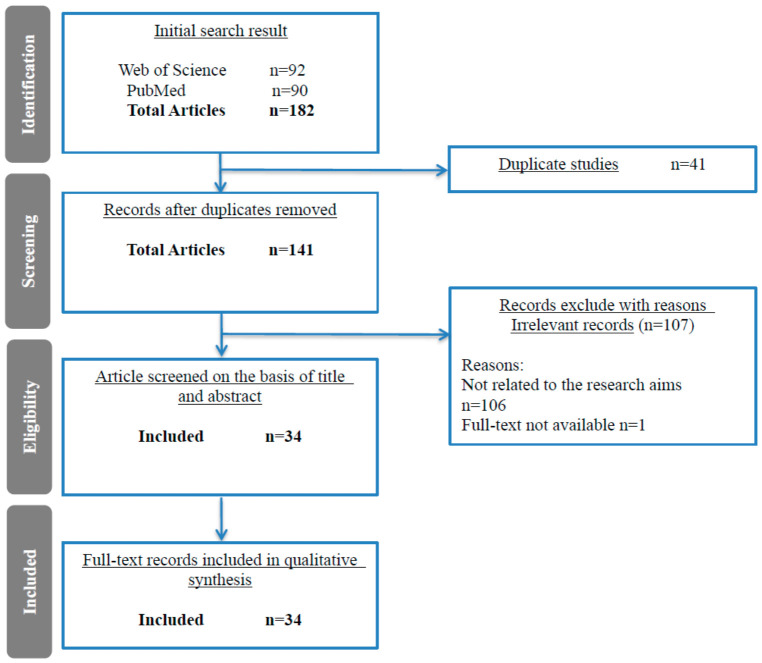
Flow chart of the systematic literature review.

AT is a general term referring to systems that support the provision, use, and evaluation of assistive products. Assistive products include “any product (including equipment, instrumentation, and software), whether specially designed and produced, or generally available” [3]. The COVID-19 pandemic has posed major public health challenges and caused extensive disruptions to AT services; thus, in this study, the influencing factors were systematically searched, screened and synthesized to help analyze and make decisions via MCDM. The first step involved conducting a preliminary data search using the keywords “Assistive Technology” and “Pandemic”, setting the conditions of only original articles published between 2019 and 2023. Two professional domain databases, Web of Science (92) and PubMed (90), were searched, yielding 182 preliminary relevant articles. After 41 duplicate articles were excluded, 141 relevant articles remained. Then, 106 articles unrelated to the use of AT during the pandemic were excluded. Of the remaining 35 articles, 1 full text was excluded, considering that the title and abstract of the article were incomplete. Finally, 34 studies related to the current objectives were identified through full-text screening, as shown in Figure 1. According to [3], service disruption, inadequate policies and systems, insufficient emergency preparedness, and limitations associated with the COVID-19 pandemic have affected the use and provision of AT. Technology is the main factor influencing the delivery of AT; therefore, on the basis of this framework, 34 related studies were analyzed to determine the possible factors affecting the interruption of AT services, and the primary factors were categorized into three dimensions: “safe delivery of AT services”, “use of Digital Assistive Technology (DAT) services”, and “development of telehealth-based AT services”.

### 2.1. Safe Delivery of AT Services

1. A1: Avoiding insufficient resources

During the COVID-19 pandemic, the need for the provision and use of AT may have been overlooked [8]. Users may have experienced interruptions in access to AT services due to inconvenience, such as the cancellation of rehabilitation courses or a reduction in personal income, which may have affected their ability to continue using paid AT services when resources are unavailable [3]. Most existing AT users experienced barriers to accessing products, treatments, and care, and AT service providers were sometimes unable to deliver existing services, resulting in challenges for AT service users [9]. The pandemic worsened access issues in low- and middle-income countries, where limited resources and inadequate government responses [10] left people with disabilities especially vulnerable. Many countries prioritized domestic healthcare systems, reducing international support for AT [3]. For example, in India, protective equipment was developed to sustain services [11]. Nepal emphasized advising stakeholders to improve access during recovery [12]. Students with disabilities also faced barriers to basic support [13]. Thus, ensuring continuity of AT services, reducing user burden, and implementing targeted emergency measures are essential for preventing future disruptions and protecting vulnerable populations. Therefore, if appropriate supporting measures or emergency response measures, such as maintaining service capacity, providing one-to-one protection measures, controlling auxiliary costs, and reducing barriers to access, are implemented to avoid a shortage of AT resources and an increase in supply-related difficulties, ensuring that the provision of AT services is not hindered or interrupted may be possible.

2. A2: Reducing policy inadequacies

Social distancing measures to control the spread of COVID-19 had significant effects on health and health equity through economic, social, and health-related behaviors as well as through disruptions to services and education [14]. Studies have shown that during the pandemic, people with disabilities were not receiving the services they needed and experienced social exclusion [15]. Social distancing and other measures taken in some countries to curb the spread of COVID-19 significantly impacted smaller-scale health resource initiatives, such as serving hearing-impaired individuals and other social minorities [6]. In addition, inadequate policies, weak systems, and a lack of emergency preparedness affected the availability of services and one-on-one support [3]. Thus, in the future, team structures can be optimized, and schedules can be adjusted to reduce contact time between AT providers and users. For example, during an infectious disease pandemic, most prosthetic service providers might adjust their assigned night shifts to reduce the burden placed on their organizations, and in some cases, providers might actively advocate for policy adjustments to support the safe resumption of AT services following the restrictive pandemic measures such as mandatory quarantines.

3. A3: Avoiding Information asymmetry

A lack of technology poses a barrier to communication. For example, during the pandemic, when hospitals experienced a shortage of hand sanitizers during the early stage, AT service providers were informed that they should wash their hands instead of using hand sanitizers. Furthermore, there were contradictory guidelines regarding the use of masks and the type of masks to be used, and a delay in the perception of messages was also noted [7]. Various other factors, such as noisy device alerts, busy medical teams with reduced interaction time with patients, and the use of personal protective equipment to mask faces that distorted sounds, also contributed to communication challenges [16]. During the pandemic, relevant news and information were constantly updated, and rapidly accessing this information was crucial. However, individuals with hearing impairments were facing difficulties in keeping up with news updates on television and radio [5]. To reduce misinformation among vulnerable groups, it may be necessary to be more inclusive in the messaging process by, for example, adding sign language and using various channels, including television, radio, newspapers, mobile apps, and landlines [17]. A government that can prepare in advance can ensure more effective delivery of AT service messages than a government that cannot prepare. However, effective preparation requires the direct involvement of individuals and their families who need AT services to ensure that these services meet their needs [11].

4. A4: Changes in users’ lives during the pandemic

The pandemic changed the lifestyle of most people, particularly elderly people, who were at a high risk for COVID-19 and were advised to avoid social interaction, but such measures often had physical and psychological effects on older people, especially those who were unfamiliar with 3C devices, such as video conferencing devices used to contact family and friends [18,19]. By contrast, the need for AT services was relatively low among visually impaired people who were at home, as they had family members to assist them [20]. In addition, access to and provision of AT services became more difficult with the implementation of changes such as reduced face-to-face communication, increased electronic communication, and increased time spent at home. For example, in people with communication impairments, access to healthcare differed and was exacerbated by COVID-19 [21]. Therefore, it is necessary to consider how to ensure the safe delivery of high-quality AT services during infectious disease outbreaks.

### 2.2. Use of Digital AT Services

1. B1: Adopting DAT Services

The pandemic had limited the use of existing AT services, and most parents and adolescents were aware of the possible delays and difficulties in accessing healthcare services during the quarantine period [22]. To effectively address the continuous delivery of AT services during the pandemic and ensure the uninterrupted provision of AT, providers had to quickly adjust their mode of providing AT services when the government permitted them to continue service provision [7]. Channels had to be established to ensure smooth access for users and solve problems such as transportation. When virtual courses were provided, access to services by users was no longer limited by traffic or distance, which also allowed users from outside the service area to access the services [23]. Consistency between digitalization and AT was encouraged to ensure the provision of AT, which made the delivery process of new AT services more complex and challenging [9]. Owing to the restrictions on transportation during the pandemic, users who needed AT services or support had to receive them at home through digital platforms, which created an opportunity for speech-language pathology professionals. They could learn about the interactions between children with disabilities and their parents, gain information about the users’ home environment, and offer support by providing semester video conferencing or recording videos of children receiving speech therapy, which could help produce positive outcomes [24].

2. B2: Accessing the support of DAT services

In the absence of technology, accessing COVID-19 information was a major challenge, and owing to the lack of service providers, many users expressed difficulty accessing new ATs. Therefore, users had to use only existing ATs, which could be offered remotely. However, providers and users both were required to have a certain level of technical expertise [3]. Providers should have the ability to teach users how to ensure product safety, and repair and maintain assistive devices; be able to establish dedicated communication channels with users; and assign specially trained staff to communicate with users. AT service providers must also be able to lend user equipment previously used for training on a long-term basis. Digital platforms also allowed users to have access to virtual services and participate in other social and therapeutic activities remotely [7]. AT products also aid decision-making, accessing prescriptions, and training [9]. Tele-rehabilitation maintains health and reduces participation barriers for users with movement disorders [22]. Supporting digital technologies facilitates population-level service delivery [25]. Future efforts should improve such technologies [24] and promote digital access through information literacy initiatives in education [26].

3. B3: Establishing an ecosystem of DAT services

Technology providers play a crucial role in delivering AT services. Technical challenges, such as a lack of smartphones and smart devices, intermittent internet connections, or poor internet connectivity, remain a major barrier [7]. However, the COVID-19 crisis created opportunities for service providers to sustain existing digital service delivery and expand it to accommodate challenging scenarios such as quarantines and social distancing, making it more inclusive and beneficial for all [27]. The existing healthcare system infrastructure, technological advancements, and effective task allocation should be leveraged to improve access to AT services [28]. However, the application of low-cost distributed virtual intelligent system interfaces may be a feasible countermeasure to address cost and accessibility challenges [29]. As a result, the pandemic has accelerated the demand for digital technology, and DAT service platforms have shared value and offered users better products and services, with users providing feedback and sometimes participating as service providers.

### 2.3. Developing Telehealth AT Services

1. C1: Innovating robust telehealth AT systems

An online service approach can continue to be adopted after the pandemic to reach a more diverse and larger group of people, use online services, and reduce the number of face-to-face transmissions. However, it also requires continued investment in information technology infrastructure and an openness to experiment with new delivery models [7]. During the development of these strategies and technologies, the rights of people with disabilities need to be prioritized, as this is a critical success factor for the adoption of these newly developed systems [3,30,31]. Additionally, through the use of digital technology and the internet, digitally assisted delivery can reduce the possibility of errors, focus on the needs of users, help detect and diagnose COVID-19 and other related problems and symptoms through the combination of technology and medical care, increase the convenience of obtaining medical resources and save costs and manpower while tracking patient conditions and reducing the risk of infection by reducing the number of patient visits to hospitals and clinics to avoid infection [32]. AT participation and feedback from service users are also considered indispensable, and to understand the needs of users and the development of services, and to increase reliance on digital technologies, it is necessary to rapidly increase the technical capabilities of telehealth [3]. Therefore, some studies suggest that mHealth should be continuously studied to effectively improve the health and quality of life of people with disabilities [33].

2. C2: Increasing the training capacity of telehealth AT providers

The implementation of AT services in different ways requires the use of different technologies for evaluation and planning, as well as changes in the delivery of AT. People could provide support and training remotely if they already had the internet, equipment, software, and access methods and were proficient, but this was often not the case [3]. When new AT services are introduced, the telehealth model has limitations in terms of user assistance [9]. For the new treatment modality to be effective, the provider of AT services (e.g., physicians) must receive appropriate training on how to use the telehealth platform and appropriate guidance on which patients are candidates for telehealth counseling [34]. In conclusion, the pandemic has highlighted the importance of the relationships among providers, users, and facilitators of AT services. While training AT service providers, it is important to integrate collaboration with facilitators (e.g., parents) in theory and practice, as they can adapt to technological needs while creating an atmosphere of communication that fosters partnership [35].

3. C3: Guiding the development of AT services for telehealth

To ensure the continuity of AT services and facilitate their remote delivery, it is essential to have a flexible combination of funding, institutions, and policies. This approach will not only support the provision of alternative services but also advance the development of relevant technologies, skills, and legislative frameworks, as well as integrate these services into health registries and training programs [3]. Efforts to leverage experience regarding the health and quality of life of people with disabilities may significantly improve underserved and underrepresented situations [36]. It is predicted that the ongoing pandemic will continue to present challenges that could hinder or delay recovery. Therefore, the most important purpose of developing a strong national policy to integrate telehealth rehabilitation into planning is to reduce the burden on patients, their families, and society and to take advantage of the increasing availability of technology and the connectivity of data to expand the growing interest in AT services and increase the scope of telehealth rehabilitation to a new level [4].

Through the literature review mentioned above, the factors that influence AT services were categorized; however, it was also found that the factors influencing AT service interruptions during the COVID-19 pandemic might be interrelated. For example, during the pandemic, emergency policy responses were inadequate, and supply providers faced shortages of safety protective equipment. Users did not receive one-on-one AT services, leading to a decline in personal economic income [3]. Communication barriers emerged due to technological shortcomings, such as the early shortage of hand sanitizers in hospitals and conflicting guidelines regarding mask usage and face coverings. The delay in conveying these messages further exacerbated the situation [7]. Policy measures for home isolation should leverage existing healthcare infrastructure, promote technological advancements, and effectively allocate tasks to ensure that AT services reach those in need. Establishing dedicated communication channels with users and assigning specially trained staff to teach users how to maintain product safety are critical steps. The repair and maintenance of assistive devices are essential for connecting users with AT service providers [7]. For new treatment modalities to be effective, AT service providers must receive appropriate training on using telehealth platforms and guidance on which patients are suitable for telehealth counseling [34]. The published studies only provided a few influencing factors of AT services or a single relation between factors. There is still an academic lack of a comprehensive discussion about the relationships among all factors of AT services and an effective filter for focusing on the critical factors of AT service. Hence, this study categorized the AT service criteria by reviewing the literature in Table 1 and determined the CSF of AT services while simultaneously considering the interactions among the criteria.

## 3. Methodology

**Figure 2:**
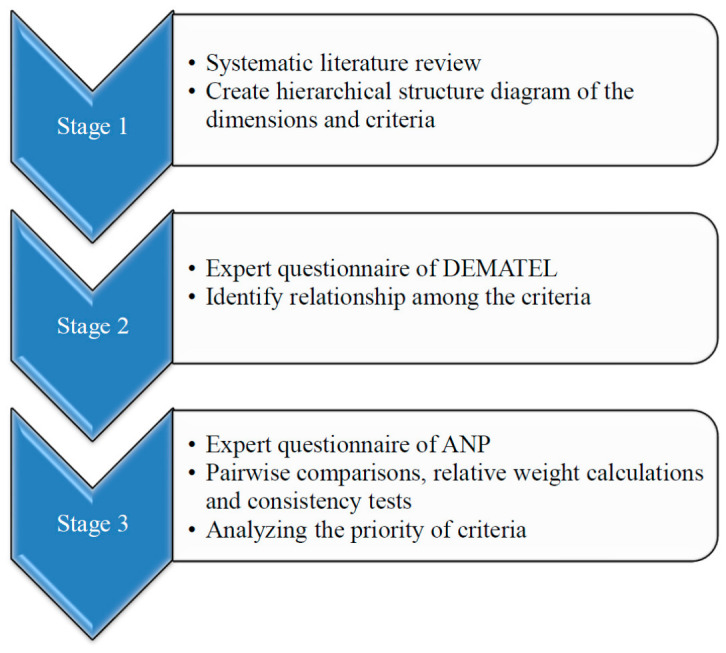
Research process.

In this study, the factors influencing AT during the pandemic were explored according to the research process (Figure 2). The research process in this study included three stages. First, a systematic literature review was performed to determine the dimensions and criteria of the MCDM model (Table 1). The MCDM method was subsequently used to construct criteria to prevent AT services from being interrupted in the post-pandemic era. DEMATEL was used in the second stage to determine the causal relationships between the criteria of AT services. Through the DEMATEL analysis of the overall relationships among factors, structural relationships can be better clarified, and this approach is an ideal method for solving complex problems [37]. At the final stage, the ANP approach was used to identify CSFs for making the best decisions. The analytic hierarchy process (AHP) is also a multicriteria evaluation method, but AHP assumes that hierarchical factors must be independent of other hierarchical factors [38]. ANP can construct different structural forms according to different problem types and can systematically address the concept of problems with dependence and feedback [39]. The detailed steps of the MCDM model, which combines DEMATEL and ANP, are as follows:

- STEP 1: Define the relationships among the factors.

Through the DEMATEL analysis of the overall relationships among factors, structural relationships can be better clarified, and complex problems can be solved [37]. To identify the relationships among the factors, this paper employed the DEMATEL questionnaire. The three definitions and the 10 criteria categorized from the systematic literature review were used to analyze the critical success factors that facilitate the prevention of AT service disruption (Table 1). The DEMATEL questionnaire was designed to compare the relationships among dimensions and criteria in pairs. The scale is used to determine the degree and direction of the interactions among the dimensions and criteria—0 is no impact, 1 is low impact, 2 is medium impact, 3 is high impact, and 4 is very high impact. The experts were divided into four groups: (1) six hospital managers (a nursing supervisor, a director of the rehabilitation department, a physical therapist leader in the department of nursing homes, and three managers in day care centers), with an average management experience of 25.5 years; (2) five who utilize AT services (three senior social workers and two physical therapists), with an average service experience of 7.2 years—the senior social worker is the hospital administrative staff who is responsible for receiving the contact point for the identification and dispatch of the health center, and the physical therapist is the hospital professional and technical personnel who provides daily life training, barrier-free assistive device assessment and home assessment for the disabled according to the International Classification of Functioning, Disability, and Health (ICF); (3) three providers of assistive devices, with an average of 12.6 years of service—sales or rental services were provided for business specialists of medical assistive device companies and sellers of medical supply stores; and (4) three users of AT services, with an average of 13.3 years of experience using assistive devices and AT services. For long-term users of assistive devices and AT services, personal mobility aids such as wheelchairs and crutches were used. The assistive devices were purchased from manufacturers. A total of 17 experts were invited to serve as expert panel scorers. Decision theory focuses on the professional judgment and consistency of experts rather than the statistical representativeness of data, so the number of expert questionnaires does not require a huge sample like general statistical surveys [38]. The consistency of expert responses is more important than quantity [39]. If the expertise of experts is sufficient and highly consistent, 5 to 15 valid questionnaires are usually sufficient in the previously published similar studies by MCDM methods [37]. This study did not involve human experiments or participants’ data. When the expert panel received invitations, they provided informed consent and voluntarily joined this study. They were only asked to identify the interactions among dimensions and criteria. Given that no human experimentation was involved in this study, the study was conducted in accordance with the guidelines of the Declaration of Helsinki, and an ethical committee protocol was not required.

- STEP 2: Calculating the direct/indirect relationship matrix.

On the basis of the DEMATEL questionnaire results answered by the experts, the correlation between factors was expressed by a matrix (Equation (1)). Afterward, the column vector and the largest value were used as the normalization base, and a normalized direct relationship matrix was calculated using Equation (2).


(1) $$X=\left[\begin{array}{cccc} 0 & x_{12} & \cdots & x_{1n}\\ x_{21} & 0 & \cdots & x_{2n}\\ \vdots & \vdots & \ddots & \vdots \\ x_{n1} & x_{n2} & \cdots & 0 \end{array}\right]$$

(2) $$\lambda =\frac{1}{\underset{1\leq i\leq n}{Max}\left(\sum_{j=1}^{n}X_{ij}\right)}$$

The normalized direct correlation matrix *N* can be calculated by Equations (1) and (2), with the direct correlation matrix X multiplied by the *λ* value (Equation (3)).


(3) N=λX

After the normalized relationship matrix was obtained, the direct/indirect relationship matrix *T* was established using Equation (4).


(4) $$T=\underset{\kappa \to \infty }{\lim}(N+N^{2}+\cdots +N^{\kappa })=N{\left(I-N\right)}^{-1}$$

- STEP 3: Calculate the prominence and the relation.

Equations (5) and (6) were used to calculate the total intensity of the affecting ($D_{i}$) and the total intensity of the affected ($R_{j})$. Then, the prominence (D+R) is obtained for each dimension/criterion, which represents the total influence of this dimension/criterion. The relation (D−R) is defined as the degree of causation, which indicates the degree of difference between the influences of these factors; if the value is positive, then the dimension/criterion is defined as the cause, and if it is negative, then the dimension/criterion is defined as the result.


(5) $$D_{i}=\sum_{j=1}^{n}t_{ij}(i=1,2,\cdots ,n)$$

(6) $$R_{j}=\sum_{j=1}^{n}t_{ij}(j=1,2,\cdots ,n)$$

- STEP 4: Establishing causal diagrams

The mutual prominence and the degree of causation between criteria and the values in the total relation matrix were presented through the setting of values greater than or equal to the threshold value. The threshold value is based on the third quartile (Q3) proposed by [40]. Other values greater than or equal to the threshold value are selected to indicate a more significant causal relationship. When the causal diagram was plotted, the prominence (D+R) was considered as the horizontal axis, and the relation (D−R) was considered as the vertical axis. To present a significant causal relationship, influence arrows can be drawn according to the setting of the threshold value, and the complex causal relationship can be simplified into an easy-to-understand visual structure. By integrating the relationship with feedback into the analysis model, Saaty [38] proposed that the ANP can construct different structural forms according to different problem types and can systematically address the concept of problems with dependence and feedback. After the precise internal relationships and interactions among targets, dimensions and criteria are predicted through the evaluation scale, the interactions between dimensions and criteria can be graphically presented to provide a complete decision-making structure. The influence relationships were chosen and displayed by an arrow in the impact digraph map.

- STEP 5: The ANP questionnaire.

According to the relationships identified through the DEMATEL, the ANP questionnaire, which compared the dimensions and criteria in pairs, could be processed. The score was determined by the panel of 17 experts. Experts had to compare the importance of every pair of criteria. The scale is used to determine the degree of mutual weight between the dimension and criteria, where 1 to 9 are the standards for the filling criteria, 1 is equally important, 3 is slightly important, 5 is quite important, 7 is extremely important, 9 is absolutely important, and 2, 4, 6, and 8 are the intermediary values of the adjacent scales.

- STEP 6: Make pairwise comparisons.

According to the experts’ scores of the relative importance of the criteria, the eigenvectors of each pairwise comparison matrix were finally calculated in the same manner as those in the analytical hierarchy program. Using Equation (7) to generate the group priority matrix A, the factors of dependence were compared in pairs, and the priority weights were generated. However, when experts answer multiple pairwise comparisons, owing to the different importance of each level, inconsistent contradictions in judgment or unclear questions may exist, leading to arbitrary answers; thus, this situation can be avoided by recalling the questionnaire [39]. The proposed consistency ratio (CR) is used to define Equation (8). CR is the ratio of the consistency index (CI) value to the random index (RI) value, which is determined according to the order of the pairwise comparison matrix, and comparison criterion *n* and its relative RI value of each level are random indices. To comply with the consistency requirement, the CR value should be less than or equal to 0.1 when comparing the aspects and criteria of the evaluation system in compliance with conformity verification. In this study, all the CR values were less than 0.1; that is, the results were consistent.


(7) $$A=\left[\begin{array}{cccc} 1 & A_{12} & \cdots & A_{1n}\\ 1/A_{12} & \cdots & \cdots & A_{2n}\\ \vdots & \vdots & \ddots & \vdots \\ 1/A_{1n} & 1/A_{2n} & \cdots & 1 \end{array}\right]$$

(8) $$\mathrm{CR}=\frac{\mathrm{CI}}{\mathrm{RI}}$$

- STEP 7: Obtain the weights.

The unweighted supermatrix represents the weights obtained from the original pair comparison and then forms the weighted supermatrix after standardization, which represents the weights of the same element in the unweighted supermatrix multiplied by the weighted number of related clusters. The supermatrix limit operation is integrated to form the limit supermatrix according to the convergence value presented, which is the corresponding weight of each criterion, and the final relative priority of each criterion is obtained.

## 4. Results

### 4.1. Results of the DEMATEL

The direct/indirect relationship matrix *T* of the three dimensions is shown in Table 2 (Columns 1 to 4). D, the total intensity of the affecting is the sum of the columns of matrix *T* and R, and the total intensity of the affected is the sum of the rows of matrix *T*. The prominence (D+R) and the relation (D−R) are calculated from the sum of the rows and columns of the total influence matrix in Table 2. Ranking by prominence (D+R) shows that “developing telehealth AT services (C)” is the most prominent (39.87). This finding indicates that “developing telehealth AT services (C)” had more influence among dimensions for maintaining AT services in the post-pandemic period. With respect to the degree of influence (D−R), “safe delivery of AT services (A)” has a positive value of 1.26, whereas “adjusting the digital AT service process (B)” and “developing telehealth AT services (C)” have negative values. This finding suggests that “safe delivery of AT services (A)” would be the dimension that could influence AT service directly and indirectly by affecting the other dimensions “use of DAT services (B)” and “developing telehealth AT services (C)”.

The direct/indirect relationship matrix *T* of the ten criteria is shown in Table 3 (Columns 1 to 11). In terms of prominence (D+R) in Table 3, “increasing the training capacity of telehealth AT providers (C2)”, “guiding the development of telehealth AT services (C3)”, and “adopting DAT services (B1)” are the top three prominent factors. In terms of the causal relationship (D−R) influence degree, “avoiding insufficient resources (A1)”, “innovating robust telehealth AT systems (C1)”, “adopting DAT services (B1)”, “reducing policy inadequacies (A2)”, and “increasing the training capacity of telehealth AT providers (C2)” all have a positive influence, so they would be regarded as the cause. Meanwhile, “avoiding insufficient resources (A1)” had the highest value of (D−R), so it would not only influence AT service, but also affect mostly other criteria to influence AT service indirectly in the post-pandemic period.

**Figure 3:**
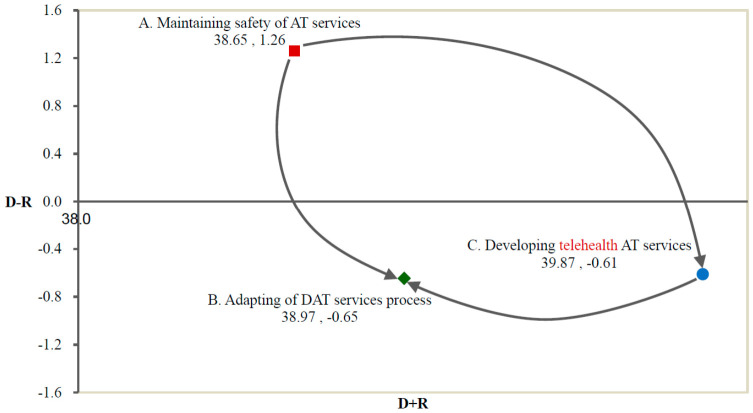
The impact-digraph map of dimensions. Note: ■ A: safe delivery of AT services; ◆ B: use of DAT services; ● C: developing telehealth AT services; and 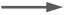 unidirectional influence.

**Figure 4:**
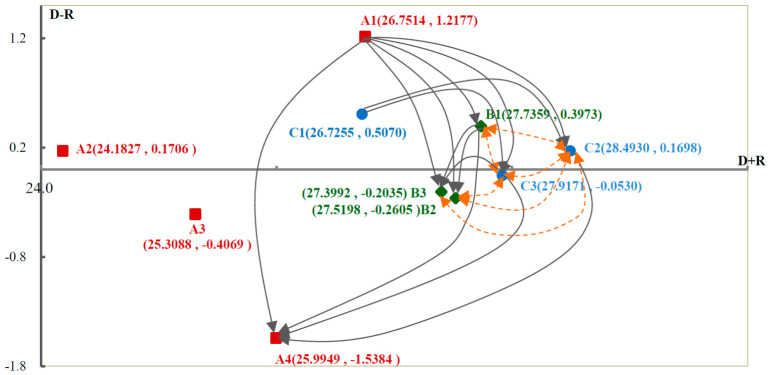
The impact-digraph map of the criteria. Note: ■ expressed as A = safe delivery of AT services; A1 = avoiding insufficient resources; A2 = reducing policy inadequacies; A3 = avoiding information asymmetry; and A4 = changes in users’ lives during the pandemic. ◆ expressed as B = use of DAT services; B1 = adopting DAT services; B2 = accessing the support of DAT services; and B3 = establishing an ecosystem of DAT services. ● expressed as C = developing telehealth AT services; C1 = innovating robust telehealth AT systems; C2 = increasing the training capacity of telehealth AT providers; C3 = guiding the development of telehealth AT services. 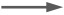 Unidirectional influence; 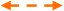 two-way interdependent; D+R is the prominence, and D−R is the relation.

To present a significant causal relationship among the dimensions and criteria, the threshold value of this study is set to the third quartile (Q3) as suggested in a previous study [40]. Accordingly, the threshold for the direct/indirect relationship matrix *T* of the dimensions is 6.73, and for the criteria, it is 1.43. Values exceeding these thresholds are in bold and shadowed in Table 2 and Table 3 to highlight significant correlations and are drawn on the causal diagrams in Figure 3 and Figure 4. The prominence (D+R) is plotted on the horizontal axis, while the influence (D−R) is plotted on the vertical axis. The data in Figure 3 indicate that “safe delivery of AT services (A)” significantly affects “use of DAT services (B)” and “developing telehealth AT services (C)”. “Safe delivery of AT services (A)” is classified as the cause, demonstrating high correlation and impact. By contrast, “use of DAT services (B)” and “developing telehealth AT services (C)” are the results, suggesting that they can be enhanced by improving the primary and secondary factors. The parameters “avoiding insufficient resources (A1)”, “adopting DAT services (B1)”, “increasing the training capacity of telehealth AT providers (C2)”, and “guiding the development of telehealth AT services (C3)” are the causes and play significant roles in Figure 4. Mutual influences are indicated by dotted double arrows, showing relationships among criteria such as “adopting DAT services (B1),” “accessing the support of DAT services (B2)”, “establishing an ecosystem of DAT services (B3)”, “increasing the training capacity of telehealth AT providers (C2)” and “guiding the development of telehealth AT services (C3)”.

### 4.2. Results of the ANP

Following the DEMATEL analysis conducted to understand the complex causal relationships between the dimensions and criteria, we employed the ANP to calculate the quantitative weight of each criterion. The results of the ANP dimension and criteria weights and rankings are shown in Table 4. The dimension/criterion with a higher weight would be more important. The dimension with the highest weight would be “Developing telehealth AT services (C)” with 51.16% and the top three criteria would be “Increasing training capacity of telehealth AT providers (C2)” with 39.61%, “Accessing supports of DAT services (B2)” with 17.53% and “Establishing ecosystem of DAT services (B3)” with 13.23%. The sum of the weights of the top three criteria is already more than 70%. As the aim of this paper is to find CSFs to avoid the disruption of AT services in the post-pandemic era, the MCDM model in this study emphasizes the interactions among the criteria, thereby highlighting the critical factors. The results showed that the ANP weights were concentrated on some criteria and that some criteria had almost zero ANP weights.

### 4.3. Discussion

In the DEMATEL, the prominence (D+R) analysis performed by the expert group revealed that “Developing telehealth AT services (C)” is the dimension with the greatest total degree of influence, where a high degree of influence represents a high correlation. “Increasing the training capacity of telehealth AT providers (C2)” is a highly correlated criterion; regarding the relation (D−R) values, the criterion “Safe delivery of AT services (A)” in the dimension “Avoiding insufficient resources (A1)” was the main influencing factor for AT service disruption during the pandemic. Based on the ranking of ANP weights, the top three CSFs are “increasing the training capacity of telehealth AT providers (C2)”, “accessing the support of DAT services (B2)”, and “establishing an ecosystem of DAT services (B3).” The pandemic had a significant effect on AT services in countries worldwide, and this study focused on the top three main CSFs and resources for preventing AT service disruptions in the post-pandemic era to achieve greater than 70% benefits.

With the “increasing the training capacity of telehealth AT providers (C2)” (39.61%) as the main critical success factor and with the speed of digital transformation, it is necessary to train providers, suppliers, or nurses to support telehealth AT services through communication channels to ensure that users can resolve technical problems and their anxiety is reduced. Therefore, talent cultivation needs to keep pace with the times. These findings are consistent with previous findings. In India, where resources are limited, a viable solution to meet the needs of users through telehealth AT services must be preceded by the education and training of community volunteers and family members, and the provision of basic AT services to users under the supervision of rehabilitation professionals [4]. In the United Kingdom, orthotists must be properly trained to understand telemedicine platforms, and users must be assessed for telemedicine consultations to ensure that this medical service is effective [34]. This approach is consistent with the results of the present study. During the COVID-19 pandemic, AT service providers and suppliers used videos or video tutorials to provide explanations, but providing further details and guidance was limited by the user’s or their family’s understanding of the explanation provided, network broadband, and 3C video operations, which impacted the overall effectiveness. In accordance with the pandemic prevention policy and the telemedicine platform established by the Ministry of Health and Welfare, medical staff must first learn the operations of the telemedicine platform and treat confirmed patients or those in home isolation using the platform. Hospitals have undergone major tests and have experience in cultivating medical personnel with telemedicine capabilities, and coupled with newly developed communication software, hospitals can provide more diverse medical services in the future.

The ANP weight for “accessing the support of DAT services (B2)” ranked second, accounting for 17.53%, indicating that providers can access the support of DAT services without contacting users, teach users how to maintain themselves and product safety, and continue to provide AT services according to the existing pandemic restrictions and model of medical services. This finding is consistent with the results of a study in the United States, which reported that with the closure of schools and clinics in the United States and worldwide, the most common solution for improving and preventing AT service interruptions during the pandemic was telemedicine [24]. According to a study of visually impaired people in India, who are at potential risk during a pandemic if they do not use digital technologies, new technologies and smartphones provide them with solutions for the problems they experience in their daily activities, improve their autonomy and safety, and encourage them to interact with the community and participate in society, thereby improving the quality of life of such visually impaired individuals [30,31]. In Taiwan, owing to the health policy and the restriction on the provision of non-emergency medical services by hospitals during pandemics, users cannot receive AT services from hospitals. They can only stay at home and experience the benefits of AT through materials and videos available online, which greatly affects users availing AT services. Furthermore, to ensure continuity of AT services, hospital professionals and technicians can follow the research experience of various countries to record simple home AT videos and develop simple assistive toolkits so that users can access AT services at home. These materials can also be used as teaching materials for future health education.

“Establishing an ecosystem of DAT services (B3)” accounted for 13.23% of the ANP weighting ranking. The pandemic has accelerated the demand for digital technology, and the ecosystem can share value and provide users with better products and services. Providers, suppliers and users are interdependent and establish supply and demand to improve operational efficiency. Previous findings were consistent with the results of the present study. According to German research, to realize the ubiquity of AT, based on policies and products available, a DAT ecosystem can be constructed through the cooperation of users, service providers, and suppliers to create an effective and accessible service delivery model that allows users and households to easily access AT services [28]. In Canada, one study revealed that reducing social interaction may affect the physical and mental health of older adults, and incorporating AT into the constructed model can effectively reduce social isolation and loneliness and increase social support and resilience among these older adults [19]. In Taiwan, severe overcrowding of hospitals was observed during the pandemic. Therefore, the Ministry of Health and Welfare, to reduce the entry and exit of people with disabilities in high-risk areas of medical institutions and prevent the spread of infection, announced that the physical and mental disability certificate of individuals can be extended to the end of the year during the pandemic period. If government policies allow the establishment of an ICF appraisal ecosystem, people with disabilities can apply for an appraisal through the online platform. The application is then received by the healthcare center, which sends the case to the responsible hospital. Subsequently, the remote video assessment and appraisal are completed, and the disabled person can apply to the Social Affairs Bureau for subsidies, or the supplier can provide suitable AT services.

Although “avoiding insufficient resources (A1)” has a low ANP weight, it is also a crucial influencing factor that cannot be ignored. Its impact is positive and belongs to the cause category, which means that the improvement of “avoiding insufficient resources (A1)” will be linked to improved criteria in other outcome classes. This criterion refers to avoiding the limitation of AT resources and minimizing the supply–demand gap during the pandemic so that the provision of AT services is not interrupted. Providing healthcare services to lower-income areas is a major challenge in India due to the lack of appropriate infrastructure, limited human resources, lack of access to personal protective equipment, lack of thorough environmental disinfection, and increased cost of care, as well as anxiety and public prejudice against Indian society caused by COVID-19 [10]. A study in Spain revealed that health authorities could remotely monitor users and provide psychological counseling via telephone; however, in the absence of sign language professionals, deaf people could not benefit from the mental health resources available during the world health crisis. These limitations are even more pronounced, especially in healthcare settings, because of the communication barriers that deaf people experienced when professionals wore masks or personal protective equipment, which increased disparities in access to healthcare in various underresourced situations [6]. This phenomenon indicates that efficient implementation of pandemic prevention measures and provision of AT services will not be possible if resources are insufficient. In Taiwan, pandemic prevention is the first priority, and medical staff, pandemic prevention materials, and resources are allocated to the front line of pandemic control. AT service users cannot make use of services if government policies do not allow allocation of safety protection materials to providers and suppliers to continue one-to-one services, develop multiple DAT service communication channels, or allow the establishment of an ecosystem for such services, thereby ensuring users receive AT services.

In summary, in this study, the causal relationship and the degree of influence among the criteria were determined according to DEMATEL, and then, the ANP was used to determine the weight of the correlation between the criteria and rank the results. The critical success factors for preventing AT service disruptions in the post-pandemic era are, in order, “increasing the training capacity of telehealth AT providers (C2)”, “accessing the support of DAT services (B2)”, “establishing an ecosystem of DAT services (B3)”, and “avoiding insufficient resources (A1)”. For hospitals and policymakers, actionable strategies could be mentioned in terms of the following three categories: resource, equipment and process. In the post-pandemic era, hospitals must rethink that the focus of digital transformation is people and should develop plans to train medical professionals in digital and professional skills through remote medical training courses to cultivate cross-disciplinary talent. In addition, how to achieve a balance of resources in limited-resource scenarios is among the important considerations for decision-makers. In terms of equipment, to ensure that AT users can make use of AT services at all times, hospital professionals and assistive device suppliers should strive to establish more comprehensive DAT services, such as by recording simple home AT videos and developing simple assistance kits for future health education promotion. In terms of this process, to prevent the disruptions to DAT services, health governments should establish an ecosystem of DAT services, such as an International Classification of Functioning, Disability and Health (ICF) identification ecosystem, on the basis of the needs of users and invite hospitals and suppliers to join the ecosystem to create a system platform for sharing value and benefits and encourage mutual assistance and cooperation, thereby making DAT services more diversified.

## 5. Conclusions

In this study, the MCDM model was used to explore the factors needed to avoid the interruption of AT services in the post-pandemic era. We conducted a systematic literature review and then used DEMATEL and the ANP to stratify complex problems in a structured manner. This MCDM model identified the CSFs for successful AT service delivery in the post-pandemic era by using criteria scoring data collected from a panel of experts. The four CSFs identified from the research results that could aid hospital decision-makers in preventing AT service disruptions were as follows: “increasing the training capacity of telehealth AT providers (C2)”, “accessing the support of DAT services (B2)”, “establishing an ecosystem of DAT services (B3)”, and “avoiding insufficient resources (A1)”. In the post-pandemic era, hospitals might have to prioritize digital transformation and train digital and professional skills using cross-disciplinary talent cultivation plans. Providing further education for AT service personnel would be suggested to improve their medical professional ability. To prevent the occurrence of DAT disruptions, the health government needs to establish an ecosystem of DAT services based on the needs of users, invite hospitals and suppliers to join the ecosystem to create a system platform for sharing value and benefits, and achieve a balance of resources in cases in which they are limited.

In this paper, the MCDM model is first employed to investigate the CSFs for preventing the interruption of AT services in the post-pandemic era. The findings filled the research gap in the literature. In terms of practice, this study provides hospital decision-makers with a better understanding of the factors that can aid in avoiding the disruption of AT services in hospitals during a pandemic. Therefore, the study hopes to help users, providers and suppliers of AT services to achieve the greatest benefits. To avoid repeating past mistakes in future pandemics involving similar infectious diseases, increasing the telemedicine capacity of medical staff and providing complete AT services are essential priorities.

This study has the limitations of relying on expert judgments and geographic representations; thus, the generalizability of the findings to other healthcare systems is limited. Owing to the wide range of available AT types, considering a specific type of AT service was not possible; thus, the scope of future research must be narrowed to a specific type of AT or expanded to AT services in other countries. Moreover, whether the CSFs that impact diverse types of AT services or different countries are the same or different should be determined and validated using empirical or longitudinal data.

## Figures and Tables

**Table 1:** Dimensions and criteria of the MCDM model.

	Dimension/Criteria	Description	Citation
A. Safe Delivery of AT services	A1. Avoiding insufficient resources	To avoid the increase in the difficulties faced due to insufficient resources and the supply–demand gap of AT services during the pandemic, the provision of AT services will not be hindered or forcefully interrupted.	[3,8,9,10,11,12,13]
	A2 Reducing policy inadequacies	To avoid reducing the positive effects of government policies and encourage the adoption of infection control measures during the pandemic, the provision of AT services is not hindered or forced to be interrupted, and service continuity is provided.	[3,6,7,14,15]
	A3 Avoiding information asymmetry	To avoid gaps or errors in the reception of information during the pandemic, the provision of AT services is not hindered or forced to be interrupted, ensuring the maintenance of the timeliness and accessibility of service provision.	[5,7,10,16,17]
	A4. Changes in users’ lives during the pandemic	The pandemic changed users’ lives, which in turn affected access to AT services. For example, face-to-face communication decreased, electronic communications increased, and time at home also increased.	[3,6,9,14,18,19,20,21,22]
B. Use of DAT services	B1. Adopting DAT services	To effectively address the pandemic-related issues and continue to deliver AT services without interruptions, providers need to adopt DAT services.	[3,4,7,9,13,22,23,24]
	B2. Accessing the support of DAT services	Providers can obtain support for DAT services without contacting the user and teach the users how to maintain themselves and the safety of the product.	[3,7,9,22,24,25,26]
	B3. Establishing an ecosystem of DAT services	The pandemic accelerated the demand for DAT, and the ecosystem of DAT services can share value and provide users with better products and services.	[3,7,27,28,29]
C. Developing telehealth AT services	C1. Innovating robust telehealth AT systems	During the pandemic, the combination and connection of user-centered telehealth technology and medical care can increase the convenience of obtaining medical resources and save costs and labor for healthcare.	[3,7,30,31,32,33]
	C2. Increasing the training capacity of telehealth AT providers	Well-trained providers, vendors, or nurses can support telehealth AT services through communication channels to reduce users’ technical anxiety.	[3,4,9,34,35]
	C3. Guiding the development of telehealth AT services	DAT can be used to maintain the continuity of telehealth AT services. The maintenance and development of telehealth AT services in the future need to be guided.	[3,4,29,36]

**Table 2:** The direct/indirect relationship matrix *T* of dimensions (threshold value ≥ 6.73).

Dimension	A	B	C	D	R	D+R	D−R	Ranking Result
								D+R
A	6.14	**6.83**	**6.99**	19.95	18.69	38.65	1.26	3
B	6.20	6.25	6.71	19.16	19.81	38.97	−0.65	2
C	6.35	**6.73**	6.54	19.63	20.24	39.87	−0.61	1

Note: A = safe delivery of AT services; B = use of DAT services; and C = developing telehealth AT services. D is the sum of the columns of matrix *T*, and R is the sum of the rows of matrix *T*. D+R is the prominence, and D−R is the relation. The bold numbers indicate the values ≥ threshold value 6.73.

**Table 3:** Direct/indirect relationship matrix *T* of the criteria (threshold value ≥ 1.43).

Criterion	A1	A2	A3	A4	B1	B2	B3	C1	C2	C3	D	R	D+R	D−R	Ranking Result D+R
A1	1.25	1.26	1.35	**1.44**	**1.44**	**1.47**	**1.45**	1.38	**1.49**	**1.47**	13.98	12.77	26.75	1.22	6
A2	1.15	1.02	1.18	1.26	1.25	1.27	1.26	1.20	1.30	1.28	12.18	12.01	24.18	0.17	10
A3	1.19	1.12	1.12	1.29	1.28	1.30	1.29	1.22	1.33	1.31	12.45	12.86	25.31	(0.41)	9
A4	1.17	1.09	1.18	1.18	1.26	1.28	1.27	1.20	1.30	1.29	12.23	13.77	25.99	(1.54)	8
B1	1.36	1.27	1.36	**1.46**	1.34	**1.47**	**1.46**	1.38	**1.49**	**1.47**	14.07	13.67	27.74	0.40	3
B2	1.31	1.23	1.32	1.41	1.40	1.32	1.41	1.34	**1.45**	**1.43**	13.63	13.89	27.52	(0.26)	4
B3	1.31	1.23	1.32	1.41	1.39	1.42	1.31	1.34	**1.44**	1.43	13.60	13.80	27.40	(0.20)	5
C1	1.31	1.22	1.31	1.41	1.40	1.42	1.41	1.25	**1.45**	**1.44**	13.62	13.12	26.73	0.51	7
C2	1.38	1.29	1.38	**1.47**	**1.47**	**1.50**	**1.49**	1.42	1.42	**1.50**	14.33	14.16	28.49	0.17	1
C3	1.34	1.26	1.34	**1.43**	**1.43**	**1.45**	**1.45**	1.38	**1.48**	1.36	13.93	13.99	27.92	(0.05)	2

Note: A = safe delivery of AT services; A1 = avoiding insufficient resources; A2 = reducing policy inadequacies; A3 = avoiding information asymmetry; A4 = changes in users’ lives during the pandemic. B = use of DAT services; B1 = adopting DAT services; B2 = accessing the support of DAT services; and B3 = establishing an ecosystem of DAT services. C = developing telehealth AT services; C1 = innovating robust telehealth AT systems; C2 = increasing the training capacity of telehealth AT providers; and C3 = guiding the development of telehealth AT services. D is the sum of the columns of matrix *T,* and R is the sum of the rows of matrix *T*. D+R is the prominence, and D−R is the relation. The bold numbers indicate the values ≥ threshold value 1.43.

**Table 4:** Comparison of critical success factor weights in the ANP.

Dimension	Criterion	ANP Weights (%)		ANP Ranking	
Maintaining safety of AT services (A)	A1 Avoiding insufficient resources	6.49%	0.00%	3	9
	A2 Reducing policy inadequacies		0.00%		10
	A3 Avoiding information asymmetry		0.00%		8
	A4 Changes in users’ lives during the pandemic		6.49%		6
Adapting the DAT services process (B)	B1 Adopting DAT services	42.04%	11.28%	2	5
	B2 Accessing the support of DAT services		17.53%		2
	B3 Establishing an ecosystem of DAT services		13.23%		3
Developing telehealth AT services (C)	C1 Innovating robust telehealth AT systems	51.16%	0.00%	1	7
	C2 Increasing the training capacity of telehealth providers		39.61%		1
	C3 Guiding the development of telehealth AT services		11.85%		4

## Data Availability

The original contributions presented in this study are included in the article. Further inquiries can be directed to the corresponding author.

## References

[1] World Health Organization (2022). Almost One Billion Children and Adults with Disabilities and Older Persons in Need of Assistive Technology Denied Access, According to New Report. https://www.who.int/news/item/16-05-2022-almost-one-billion-children-and-adults-with-disabilities-and-older-persons-in-need-of-assistive-technology-denied-access--according-to-new-report.

[2] Global Alliance of Assistive Technology Organizations (2022). Assistive Technology Outcomes and Impact—A Global Grand Challenge. https://www.gaato.org/post/at-outcomes-and-impact-a-global-grand-challenge.

[3] Smith E.M., Hernandez M.L.T., Ebuenyi I.D., Syurina E.V., Barbareschi G., Best K.L., Danemayer J., Oldfrey B., Ibrahim N., Holloway C. (2022). Assistive technology use and provision during COVID-19: Results from a rapid global survey. Int. J. Health Policy Manag..

[4] Christy B., Mahalakshmi M., Aishwarya T., Jayaraman D., Das A.V., Rani P.K. (2022). Tele-rehabilitation for persons with vision impairment during COVID-19: Experiences and lessons learned. Indian J. Ophthalmol..

[5] Madahana M., Khoza-Shangase K., Moroe N., Mayombo D., Nyandoro O., Ekoru J. (2022). A proposed artificial intelligence-based real-time speech-to-text to sign language translator for South African official languages for the COVID-19 era and beyond: In pursuit of solutions for the hearing impaired. S. Afr. J. Commun. Disord..

[6] Recio-Barbero M., Sáenz-Herrero M., Segarra R. (2020). Deafness and mental health: Clinical challenges during the COVID-19 pandemic. Psychol. Trauma Theory Res. Pract. Policy.

[7] Puli L., Layton N., Mont D., Shae K., Calvo I., Hill K.D., Callaway L., Tebbutt E., Manlapaz A., Groenewegen I. (2021). Assistive technology provider experiences during the COVID-19 pandemic. Int. J. Environ. Res. Public Health.

[8] Naghavi A., Faramarzi S., Abbasi A., Badakhshiyan S.-S. (2022). COVID-19 and challenges of assistive technology use in Iran. Disabil. Rehabil. Assist. Technol..

[9] Ghosh R., Healy A., Prabhune A., Mallavaram A., Raju S., Chockalingam N. (2022). Provision of rehabilitation and assistive technology services in a low resource setting during the COVID-19 pandemic and introduction of telehealth: Service users’ and providers’ perspectives. Assist. Technol..

[10] Mont D., Layton N., Puli L., Gupta S., Manlapaz A., Shae K., Tebbutt E., Calvo I., Sidiqy M., Dube K. (2021). Assistive technology during the COVID-19 global pandemic: The roles of government and civil society in fulfilling the social contract. Int. J. Environ. Res. Public Health.

[11] George C.E., Inbaraj L.R., Rajukutty S., De Witte L.P. (2020). Challenges, experience and coping of health professionals in delivering healthcare in an urban slum in India during the first 40 days of COVID-19 crisis: A mixed method study. Br. Med. J. Open.

[12] Karki J.K., Rushton S., Karki A., Rijal B., Makai P., Neupane R., Joshi S., Basnet S., Bhattarai S., De Witte L. (2023). Impact of the COVID-19 pandemic on the lives of persons with disabilities in rural Nepal: A mixed method study. Public Health Pract..

[13] Aquino K.C., Scott S. (2023). Supporting students with disabilities during the COVID-19 pandemic: The perspective of disability resource professionals. Int. J. Environ. Res. Public Health.

[14] Douglas M., Katikireddi S.V., Taulbut M., McKee M., McCartney G. (2020). Mitigating the wider health effects of COVID-19 pandemic response. Br. Med. J..

[15] Toquero C.M.D. (2020). Inclusion of people with disabilities amid COVID-19: Laws, interventions, recommendations. Multidiscip. J. Educ. Res..

[16] Moreland C.J., Ruffin C.V., Morris M.A., McKee M. (2021). Unmasked: How the COVID-19 pandemic exacerbates disparities for people with communication-based disabilities. J. Hosp. Med..

[17] Vieira C.M., Franco O.H., Restrepo C.G., Abel T. (2020). COVID-19: The forgotten priorities of the pandemic. Maturitas.

[18] Bennett Gayle D., Yuan X., Knight T. (2021). The coronavirus pandemic: Accessible technology for education, employment, and livelihoods. Assist. Technol..

[19] Jutai J.W., Tuazon J.R. (2022). The role of assistive technology in addressing social isolation, loneliness and health inequities among older adults during the COVID-19 pandemic. Disabil. Rehabil. Assist. Technol..

[20] Luo G., Pundlik S. (2021). Influence of COVID-19 lockdowns on the usage of a vision assistance app among global users with visual impairment: Big data analytics study. J. Med. Internet Res..

[21] Lake B., Maidment D.W. (2023). “Is this a new dawn for accessibility?” A qualitative interview study assessing teleworking experiences in adults with physical disabilities post COVID-19. Work.

[22] Faccioli S., Lombardi F., Bellini P., Costi S., Sassi S., Pesci M.C. (2021). How did Italian adolescents with disability and parents deal with the COVID-19 emergency?. Int. J. Environ. Res. Public Health.

[23] Groszew L., Zavoda E. (2022). How one blindness agency successfully pivoted in providing essential services in the midst of a global pandemic. J. Vis. Impair. Blind..

[24] Therrien M.C., Biggs E.E., Barton-Hulsey A., Collins S.C., Romano M. (2022). Augmentative and alternative communication services during the COVID-19 pandemic: Impact on children, their families and service providers. Augment. Altern. Commun..

[25] Boggs D., Polack S., Kuper H., Foster A. (2021). Shifting the focus to functioning: Essential for achieving sustainable development goal 3, inclusive universal health coverage and supporting COVID-19 survivors. Glob. Health Action.

[26] Koob A.R., Oliva K.S.I., Williamson M., Lamont-Manfre M., Hugen A., Dickerson A. (2022). Tech tools in pandemic-transformed information literacy instruction. Inf. Technol. Libr..

[27] Pineda V.S., Corburn J. (2020). Disability, urban health equity, and the coronavirus pandemic: Promoting cities for all. J. Urban Health.

[28] Layton N., Mont D., Puli L., Calvo I., Shae K., Tebbutt E., Hill K.D., Callaway L., Hiscock D., Manlapaz A. (2021). Access to assistive technology during the COVID-19 global pandemic: Voices of users and families. Int. J. Environ. Res. Public Health.

[29] Bricout J., Greer J., Fields N., Xu L., Tamplain P., Doelling K., Sharma B. (2022). The “humane in the loop”: Inclusive research design and policy approaches to foster capacity building assistive technologies in the COVID-19 Era. Assist. Technol..

[30] Senjam S.S. (2022). Smartphone: A smart assistive device for people visual disabilities among COVID-19 pandemic. Indian J. Public Health.

[31] Senjam S.S., Primo S.A. (2022). Challenges and enablers for smartphone use by persons with vision loss during the COVID-19 pandemic: A report of two case studies. Front. Public Health.

[32] Javaid M., Haleem A., Vaishya R., Bahl S., Suman R., Vaish A. (2020). Industry 4.0 technologies and their applications in fighting COVID-19 pandemic. Diabetes Metab. Syndr. Clin. Res. Rev..

[33] Long E., Vijaykumar S., Gyi S., Hamidi F. (2021). Rapid transitions: Experiences with accessibility and special education during the COVID-19 crisis. Front. Comput. Sci..

[34] Eddison N., Healy A., Calvert S., Chockalingam N. (2023). The emergence of telehealth in orthotic services across the United Kingdom. Assist. Technol..

[35] Nissim M., Ido O., Sanduka Y., Shmerling C., Ariel N. (2023). In relation to the relationship: Teachers of pupils with multiple disabilities and parents following the COVID-19 pandemic. Eur. J. Spec. Needs Educ..

[36] Lee R.E., Suh B.C., O’Neal A., Cameron C., O’Connor D.P., Ohri-Vachaspati P., Todd M., Hughes R.B. (2023). Association of mobile health (mHealth) use with health status and COVID-19-related concerns by people with mobility impairments. Disabil. Rehabil. Assist. Technol..

[37] Hsu W., Shih F.P. (2023). Key Factors for Enhancing Home Care Workers’ Intention to Stay by Multiple-Criteria Decision Analysis. Healthcare.

[38] Saaty T.L. (1990). How to make a decision: The analytic hierarchy process. Eur. J. Oper. Res..

[39] Saaty T.L. (1996). Decision Making with Dependence and Feedback: The Analytic Network Process.

[40] Bolaños R., Fontela E., Nenclares A., Pastor P. (2005). Using interpretive structural modelling in strategic decision-making groups. Manag. Decis..

